# On Fibroids of the Uterus

**Published:** 1875-06

**Authors:** 


### On Fibroids of the Uterus: Intra-uterine Myomata.

M. Stoltz, writing in the *Revue Medicale de 'l Est,* February 1, 1875, says that pedicellated fibrous bodies, commonly known by the name of polypi, arise either from the cavity of the body or from the neck. The former are not properly pedicellated, but adhere to a more or less limited surface of the uterus; they are really sessile. They become pedicellated by the excessive uterine efforts at their expulsion. When in the vigina, they are not really pedicellated. Should the part to which they are attached offer considerable resistance, the capsule may become elongated into a veritable pedicle. As a rule, the fibrous body draws down with it the portion of the uterine wall to which it is attached, causing an inversion of the uterine parietes, allowing thereby the polypus to project into the vagina. This fact should never be absent from the mind of the operator, whether the polypus be still within the uterine cavity or protruded into the vagina, or replaced into the uterus, or drawn down by the manipulator into the vagina. The first object is to make out whether the tumour is pedicellated or sessile; if the latter, the extent of the base. Should it prove to have a wide base, he strongly recommends making a couple of incisions into the capsule with a pair of curved scissors; the tomour then peels out of its capsule as the rind does from off an orange. He has done this operation many times with success; as a rule, it is the most expeditious and least dangerous method of dealing with these growths; but he admits exceptions. The wire écraseur he objects to, on account of the danger of cutting the uterus, which has been the case more than once in the hands of the most skilled operators. Professor Braun-Fernwalt, for the same reason advises the tumour to be cut in halves, or a piece cut out of it with the galvanic wire cautery. Other authors strongly recommend that the patient should not be anæsthetized before tightening the wire of the écraseur, as, from the uterine tissue being a sensitive structure, her sensations will be a fair guide as to whether the uterus has been impinged upon or not.—*Medical Abstract.*

